# Implementing Innovative Approaches to Healthcare in a Lower-Middle Income Country: Perspectives from Malawi

**DOI:** 10.2147/IJGM.S285130

**Published:** 2020-12-31

**Authors:** Emma Larsson, Mala Mawkin, Simon D Taylor-Robinson, Peter Harrington, Hastings Gondwe, Chris Watson, Joseph Gallagher, Mark Ledwidge, Griphin Baxter Chirambo, John O’Donoghue

**Affiliations:** 1 Imperial College London, South Kensington, London SW7 2BX, UK; 2 gHealth Research Group, University College Dublin, Dublin, Ireland; 3 St John’s Hospital, Mzuzu, Malawi; 4 Wellcome-Wolfson Institute for Experimental Medicine, Queens University Belfast, Belfast, Northern Ireland; 5 Mzuzu University, Mzuzu, Malawi; 6 Malawi eHealth Research Centre, University College Cork, Cork, Ireland; 7 ASSERT Research Centre, University College Cork, Cork, Ireland

**Keywords:** eHealth, mHealth, non-communicable diseases, respiratory disease, cardiovascular disease, diabetes

## Abstract

**Introduction:**

Safe, reliable, and effective healthcare systems are essential for all nations to ensure the health and wellbeing of their citizens. However, this is not always achievable with clinical therapies constantly evolving, resulting in a domino effect of structural, policy and training changes. For low- and middle-income countries (LMICs), implementing change is restricted. It is essential that innovative and realistic solutions are developed, so that effective change can be realised in LMICs.

**Materials and Methods:**

In this report of a global health conference held in July 2019, six perspectives are presented which aim to generate long-term positive change in Malaŵi. Perspective 1: Pneumonia – the BIOTOPE study (BIOmarkers TO diagnose PnEumonia) sought to determine the aetiology of pneumonia in children presenting in primary care. It assessed blood-based markers of bacterial infection as part of a rapid diagnostic approach to better utilise existing resources in Malaŵi. Perspective 2: Cardiovascular – the CARDIA project (CARdiac Dysfunction in Africa) was established to assess clinical and biochemical phenotypes of diabetic patients in Malaŵi. Perspective 3: Asthma – an observational study was conducted to implement a health system strengthening initiative for asthma. The use of locally adapted formularies and protocols with ongoing online mentoring through expert partnerships provided an opportunity to sustainably build capacity. Perspective 4: Sustainable Partnerships – establishing the Malaŵi electronic Health (eHealth) Research Centre, an international hub to develop education, research and innovation for long-term collaboration. Perspective 5: Part-Time PhD Studies – undertaking a part-time PhD within a LMIC provides logistic challenges, but also a number of opportunities for observational research. Perspective 6: Medical electives – an undergraduate elective allows real exposure to global health and facilitates life-long collaborations at an early stage in a medical career.

**Conclusion:**

Malaŵi is an under-doctored and resource-poor country. North-South partnerships in Malaŵi should be strengthened with particular emphasis on healthcare innovations, such as eHealth, which allow healthcare problems to be highlighted early while preventative measures are still possible.

## Introduction

Malaŵi is an independent, democratic republic in South-Central Africa. Formerly known as Nyasaland, it gained independence from the British in 1964 and was subject to years of a one-party state under the first President of the country, Dr Hastings Banda. His later years were marked by economic mismanagement and corruption and economic growth slowed to a halt, having initially been promising in the earlier years of independence. Densely populated with close on 20 million people, Malaŵi consistently ranks as one of the poorest countries in Africa. However, it is resource rich, straddling one of Africa’s largest lakes, Lake Malaŵi.^1^

The country is beset by poor healthcare management, a lack of skilled medical manpower and a lack of investment in healthcare infrastructure. The problems are magnified by a medical “brain-drain” to the countries of the North and to the Arab countries of the Gulf region. Malaŵi has one of the lowest life expectancy rates in the world, accompanied by one of the highest rates of infant mortality. On this background, traditional health issues, such as malaria, schistosomiasis, childhood infections and tuberculosis remain significant problems, but in a world of easy communications, where traditional, more healthy diets are changing towards westernized, low-fibre, fast-food diets, non-communicable diseases such as hypertension, coronary artery disease, stroke and diabetes are becoming much more prevalent.

We report the proceedings of a conference on Healthcare in Malaŵi, held in Cork, Ireland on 5th July 2019, which brought together several stakeholders active in Malaŵi who are attempting innovate practical solutions to the country’s healthcare impasse. The rationale was to allow those involved in the healthcare dialogue in Northern Malaŵi to learn from the experience of each other and to allow thoughts to be shared and to bring about discussions on effective sharing of resources for the future. Each of the perspectives discussed below is a transcript of an oral presentation given at the conference (Table 1). The scientific rationale for the research studies was to identify disease patterns in a resource-poor setting using either low-cost biomarkers or e-Health assessment.

**Table 1:** Perspectives

Perspective Number	Projects Discussed as Oral Presentations at the Global Health Meeting, Cork, Ireland, July 2019
Perspective 1	Childhood pneumonia in Malaŵi
Perspective 2	Diabetes-associated cardiac dysfunction in Malaŵi
Perspective 3	Asthma Management in Malaŵi
Perspective 4	eHealth project development in Malaŵi
Perspective 5	Postgraduate education in Malaŵi
Perspective 6	Student medical electives in Malaŵi

## Perspective 1: Childhood Pneumonia in Malaŵi – A Different Disease in the Era of Extended Immunisation?

Despite recent advances in reducing childhood mortality, pneumonia remains a major cause of morbidity and mortality among children under 5 years of age, with approximately 1 million deaths each year.^2^ Allied to this is the growing concern regarding antimicrobial resistance driven by overuse of antibiotics.^3^,^4^ The increased use of universal immunisation, including *Haemophilus influenzae* type B (Hib) and pneumococcal vaccines are likely to have altered the proportion with viral illness and the clinical profile of this illness. For instance, a recent study has shown that, in Malaŵi, the number of children with non-severe, fast-breathing pneumonia who needed amoxicillin treatment for one child to benefit was 33 children^5^ suggesting a large prevalence of viral illness in the majority. Very few studies have been conducted in primary care,^6^ despite this being the place where pneumonia is most likely to present.

The use of microbiological techniques for diagnosis is hampered by the high rate of carriage of bacteria and viruses in children^7^ and lack of technology in LMICs. The PERCH (Pneumonia Etiology Research for Child Health) study undertook a large study in seven African and Asian countries and found that only a small group of pathogens was responsible for most cases of childhood pneumonia needing hospital admission.^8^ The PERCH study itself was not used as a template for further work in Malaŵi. Although useful at a population level, other approaches are needed to identify bacterial pneumonia pathogens for individual children. The use of host response biomarkers to identify those with bacterial infections may be a useful tool in this regard.

The markers of severity may also be changing. Recent work^9^ has shown that 39% of fatal cases of pneumonia were defined as having non-severe pneumonia, requiring only home treatment. The fact that the World Health Organisation (WHO) criteria would have discharged these children with oral antibiotics highlights the need for new markers of severity as these are used to guide primary healthcare workers as to who should be referred to hospital.

The BIOTOPE study (BIOmarkers TO diagnose PnEumonia) was a prospective cohort study in Malaŵi which sought to determine the aetiology of community-acquired pneumonia in children presenting in primary care in Northern Malaŵi and identify predictors of children requiring hospitalisation. Research undertaken has received prior ethical approval by Mzuzu University Ethics Committee.

The BIOTOPE study also undertook pilot exploratory work to identify possible blood-based markers of bacterial infection. Its results are awaited, but provide an example of useful North-South collaboration in a remote region of Malaŵi.

In summary, the study is expected to show the utility of prospective biomarkers in highlighting those children who may need future hospital care. We would suggest that this work will show the usefulness of low-cost biomarker development in resource-poor countries with great potential to save lives. We would recommend that funding bodies are open to further low-cost biomarker development in the Global South.

## Perspective 2: The CARDIA Project – Cardiac Dysfunction in Africa

It is currently estimated that 415 million people worldwide suffer from diabetes with more than 80% living in LMICs. Sub-Saharan Africa will see a rise in incidence of diabetes from 14.2 million in 2015 to 34.2 million in 2040 (IDF 2015). Overall, Africa will undergo an approximate relative increase of 140%, the largest in the world. Just like the developed world, 90% of diabetes patients in sub-Saharan Africa have Type 2 diabetes mellitus (T2DM).

The most common cause of death in people with T2DM is cardiovascular disease. Cardiovascular disease is on the rise also, as it is estimated that the number of people with hypertension in Africa will increase by 68% from 75 million in 2008 to 126 million in 2025. A recent review demonstrated that in sub-Saharan Africa, coronary heart disease may affect 5% to 8% of patients with T2DM, and cardiomyopathy up to 50% of patients, which is very different to patterns in Western countries.^10^

The continent is at a tipping point, where public health action must take place now in order to address the startling increase in diabetes and cardiovascular disease. Current efforts to diagnose and treat diabetic patients in Malaŵi are in a primal state. The HIV and TB pandemic soaked up much of the resources and commitment of government and aid agencies over the past two decades. Education and resources for diabetic prevention and treatment need to be developed and strengthened to prevent and treat T2DM and its associated complications.

In the year 2000, the estimated prevalence of diabetes among adult Malaŵians was less than 3%.^11^ A more recent survey in 2009 using the WHO STEPwise approach to chronic disease risk factor surveillance, found that 5.6% of adults in Malaŵi had fasting hyperglycaemia and further 4.2% had impaired fasting glucose.^12^ The majority of those found to have hyperglycaemia were unaware that they had diabetes. These findings indicate that the number of people living with diabetes in Malaŵi is increasing rapidly and lack of awareness makes the population very vulnerable.^12–16^ However, data on treatment and control of diabetes in Malaŵi are lacking. Turning to macrovascular complications of diabetes, diagnosis of cardiac dysfunction is a challenge as it relies on echocardiography. The cost of equipment, technical training, and the need to provide the service to mostly rural populations in Malaŵi, and other African countries make it unfeasible to provide this on a wide scale. The development of new biomarkers to identify cardiac dysfunction would reduce the need for echocardiography and allow the implementation of diagnosis and treatment at an early stage to a large population of patients with diabetes and/or hypertension.

The CARDIA project was set up in the northern region of Malaŵi, and its epicentre is in Mzuzu Central Hospital. Research undertaken has received prior ethical approval by Mzuzu University Ethics Committee. The purpose of this project is to undertake to study the clinical and biochemical phenotype of patients with diabetes in Malaŵi, including the analysis of cardiac structure and function using echocardiography and analysing circulating surrogate markers of cardiac stress. More than 300 patients have been recruited to date to the CARDIA study, and phenotypic profiling is currently underway.

In summary, the study is expected to shed light on the cardiac sequelae of diabetes in Malaŵi and garner aetiological associations which may be useful in future preventative strategies in the country. We would recommend that further observational studies are undertaken to combine with epidemiological data in order to highlight the reasons for local differences in diabetes-related morbidity and mortality.

## Perspective 3: Effects of a Multifaceted Intervention on Asthma Management in a Primary Care Setting in Malaŵi

Asthma is responsible for considerable global morbidity. It is associated with marked health-care costs in LMICs such as Malaŵi. To delineate these issues, an on-going Malaŵi-based study has sought to implement an intervention to improve asthma care for adults and children in a hospital-based clinic in Northern Malaŵi. Research undertaken has received prior ethical approval by Mzuzu University Ethics Committee.

The newly established asthma clinic is in St John’s Hospital in Mzuzu, the third-largest Malaŵian town. Often, basic supplies are not available including ventilatory spacers or peak flow meters. A multifaceted intervention was undertaken involving creation of an inhaler-centred asthma-chronic obstructive pulmonary disease (COPD) drug formulary, local clinical guidelines, structured outpatient clinical review, in-person educational workshops and online mentoring of clinical staff. A clinical officer was appointed as an in-hospital mentor, and protocol implementer - and to assist in structured outpatient clinical review. All patients attending the clinic with asthma in the 12 months following the workshop were included.

Of the six medicines on the WHO essential medicine list only one, epinephrine, was regularly available in the current hospital formulary in Mzuzu. A locally appropriate medication list was constructed, and medication supply was addressed. One hundred and forty-seven patients with asthma-COPD attended during the 12-month study period. At time of first attendance, 101 patients were taking salbutamol tablets, 16 were on oral aminophylline and 13 were on no medication regularly. Only 17 patients used salbutamol inhalers, and none used beclomethasone inhalers. There was no regular follow-up of patients.

Following the intervention, 146 patients were on salbutamol inhalers and beclomethasone inhalers. One refused inhaled medication altogether. Forty-two patients required admission in the 6 months prior to the intervention, with four requiring readmissions to hospital. In the 12 months following the intervention, only 18 patients required admission, and none required readmission. One hundred and one patients (69%) attended the review clinic on the planned date. Twenty-one (14%) attended on a different date, mostly because they had no funds to travel on the day appointed. Twenty-five patients (17%) did not come for review.

This observational study confirms that it is feasible to implement a health system strengthening initiative for asthma in a low-income country. The use of locally adapted formularies and protocols with ongoing online mentoring in partnership with Irish primary healthcare staff provided an opportunity for capacity building in a sustainable manner. Following the intervention, the structures of care improved and data are being collected routinely on asthma control. Expanding this approach to other chronic diseases is in progress.

In summary, this study provides a good example of medical protocol development with early intervention to save lives. We believe that this example can be extended to other non-communicable diseases in Malaŵi. We recommend that funding bodies are open to such North-South collaborations in other areas of medicine.

## Perspective 4: Establishing a Sustainable North-South eHealth Research Partnership through the Development of National eHealth Research Centre – A Malaŵi Perspective

The application of Information and Communications Technology (ICT) across healthcare systems through electronic Health (eHealth) and mobile Health (mHealth) is not new. ICT solutions do come at a cost, but their benefits can be quite substantial and in most cases are the primary communication and data management backbone for the majority of healthcare services.^17^ For any country to fully utilise ICT as part of its healthcare strategy, it is essential that it has a robust foundation within the area of ICT. The WHO report,^18^ provided an ICT Development Index of 125 WHO member states. Countries with advanced ICT capabilities included Iceland: 3rd, USA: 17th and Ireland: 23rd with Malaŵi ranked at 145. The Malaŵian government is currently implementing a programme^19^ to build up its national ICT capacity.

**Figure 1:**
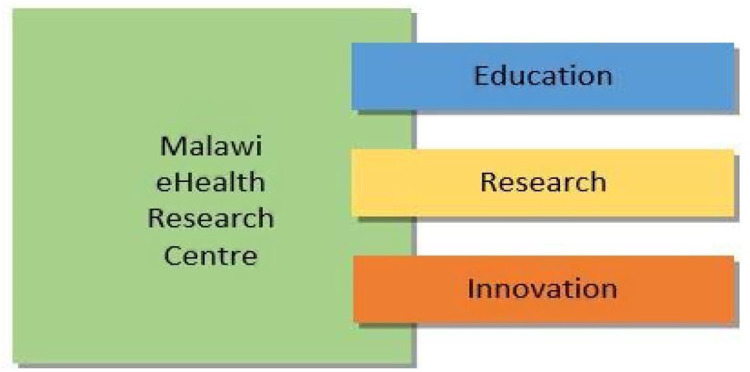
Education, research and innovation are the key building blocks of the Malaŵi eHealth Research Centre.

In 2012, University College Cork (Ireland) and Mzuzu University (Malaŵi) in partnership with the Malaŵian Ministry of Health, founded the Malaŵi eHealth Research Centre (www.ehealthmalawi.org). The centre has been “established to build up eHealth capacity across Malaŵi in the areas of education, research and innovation to enhance the effectiveness of Healthcare services.” With ICT as a central enabling theme for enhancing the delivery of healthcare services (Figure 1).

A key weakness of North-South partnerships has been that they are largely managed from outside the developing countries, and are sustainability donor-dependent.^20^ Chu et al^21^ highlighted that LMICs have to take an active role in leading or directing these research collaborations in order to maximize the benefits and minimize the harm of inherently inequitable relationships. To that end, the Malaŵi eHealth Research Centre, which is based on Mzuzu University campus, has been building ICT, eHealth, education and research capacity through its academic staff. This in turn has built up a larger national capacity through the University’s Undergraduate and Postgraduate programmes. As such, Mzuzu University academic staff members are critical to the long-term development of eHealth across Malaŵi through Education (skills development), Research (identification and development of new technologies/processes) and Innovation (implementation of new approaches to enhance healthcare delivery). This also has helped to reduce the potential for high staff turnover as highlighted by Bates et al^22^ as one of the key challenges in North-South partnership building.

The Malaŵi eHealth Research Centre has been established to enhance the effectiveness of healthcare services across Malaŵi through ICT and is built on a strong and sustainable, mutually beneficial North-South partnership. Research undertaken has received prior ethical approval by Mzuzu University Ethics Committee. Since its inception, it has adopted a participatory approach to its development and operation across all three themes: Education, Research and Innovation. Such an approach as highlighted by Huang et al^23^ provides all stakeholders with transparency regarding the importance of recognising the political, social and historical context within which information systems have to function. This platform forms the basis of a solid foundation for eHealth research-based projects to go beyond the pilot phase of research and development and make the transition into national implementation for enhanced healthcare delivery.

In summary, in a country that is resource-poor and heavily under-doctored, the development of e-Health systems allows the portent for preventative public health programs to be developed in very remote areas of the country, facilitating early disease detection while preventative measures are still possible. We would recommend that eHealth projects are developed for both communicable and non-communicable diseases in Malaŵi.

## Perspective 5: Experiences of Undertaking a Mobile Health-Based Part-Time PhD in Malaŵi

Malaŵi is a country in southern Africa with limited financial resources. The health sector is also very much affected by the shortage of staff, and of medicines, among other key resources.^24^ Hence, it is difficult for the government to successfully undertake the full implementation of its Healthcare Strategic Development Plan. As a result, clinical staff are encouraged to conduct their post-basic training on a part-time basis. University lecturers have also experienced similar problems. As a natural consequence, clinical researchers who also hold an academic post in Malaŵi face several obstacles when undergoing their PhD studies.

One of the major challenges of undergoing a mHealth PhD in Malaŵi on a part-time basis has been job responsibilities. With the shortage of staff, it means that the job distribution among lecturers to teach and provide other services to students is currently allocated evenly, without special consideration to any staff currently in training. All lecturers are required to teach in class according to the teaching schedule, follow-up with students in their clinical placement for clinical supervision and of course mark the assignments and exams given to students. This on its own takes a lot of time away from any PhD studies that the lecturer may have, albeit on a part-time basis, compromising the quality of work and potential research output aimed at bettering the quality of the Malaŵian healthcare infrastructure.^25^

Most organizations including the universities have limited financial resources to support students undergoing PhDs with such students being encouraged to use their own financial resources for training. This results in most people failing to complete their training, because they cannot manage to pay fees. Likewise, in cases where PhD candidates do not have dedicated scholarship funding or institutional support, it greatly affects basic research activities. Data collection is one example of this. Lack of financial support also affects the ability of a research student to regularly meet research supervisors, especially if some of these are far from the student’s workplace and in some instances are outside the host country.^26^

The consistent power blackouts in Malaŵi are also a major issue as part of PhD training. Sometimes the problems with electricity may affect the student’s ability to have skype calls, to access internet, and even to type assignments when required to do so. There are also some challenges with telecommunication system having limited internet access. This makes it difficult for a student to hold skype calls with their supervisors and other participants. Likewise, limited internet bandwidth restricts access to the necessary academic databases to view and download journals and papers necessary for the publication of research articles.^27^

The libraries in Malaŵi do subscribe to a variety of different journals and databases, but there are many that remain inaccessible, owing to lack of funds with consequent implications on learning for research students.

Highlighting an area of success, as part of his PhD studies, one of the authors (GBC) was part of an international collaboration, the Supporting LIFE project. Research undertaken received prior ethical approval by Mzuzu University Ethics Committee. This funding enabled him to engage with research teams all over the world: in Ireland, UK, USA, Sweden, Switzerland and Taiwan. It also provided him with an opportunity to be part of a large randomized controlled trial which was developed to evaluate the effectiveness of mHealth within a community setting in Northern Malaŵi. Through these engagements, he has assisted in building research capacity at the University of Mzuzu and more widely across Malaŵi.

In summary, North-South partnerships allow many of the funding issues faced by those undertaking higher education in Malaŵi to be overcome. By working in North-South healthcare projects alongside project development, PhD students have access to resources for academic development that are otherwise unavailable. We would recommend that the model of the Supporting LIFE project is not only useful for Malaŵi in general, but widely applicable across the Global South.

## Perspective 6: Experiences of Undertaking a Medical Elective in Malaŵi

Two final year medical students (MM and EL) travelled to Malaŵi as part of the Malaŵi eHealth Research Centre exchange programme (www.ehealthmalawi.org), visiting a variety of hospitals, clinics, mission workers, embassies and the Ministry of Health to see first-hand how data and innovation were driving change in Malaŵi’s healthcare system. However, they were faced with a wide range of realities which healthcare systems within a LMICs face on a daily basis.

They were based in St John’s Hospital in Mzuzu, where the facilities were basic and key resources were lacking. On the ward round, there were no curtains separating patients’ beds and providing privacy. The patients had to bring their own bed linen as the hospital could not afford to cover this cost. Additionally, normal hygiene protocols, such as hand washing, were not adhered to due to lack of cleaning supplies. The general theme in all hospitals was one of a lack of healthcare workers, resources and capacity. Most patients never saw a doctor, but rather clinical officers (akin to physician assistants) and other healthcare staff who complete limited training to function as a service provider. These staff members then go on to provide medical care and to operate on patients despite not having international standard qualifications to do so. Such workers function with the limited resources available to them on a daily basis. However, due to more fundamental problems out of their control, they are generally limited in what they could do. While interested in free online resources and shared education to aid their own medical education in Malaŵi, the limited availability to electricity, computers and internet affects the usefulness of these tools, as with all eHealth provision in LMICs. However, even though only 9% of the Malaŵian population are connected to the power grid,^28^ most of the hospitals are connected. Despite this, frequent power outages limit the ability to rely on electronics to study or collect data for audit and research. Moreover, internet availability is scarcer than electricity, and often not available in hospitals, hence data sharing and the potential for collaboration is impacted.

Despite these harsh realities a number of well-established organises in Malaŵi are beginning to provide the essential eHealth (desktop-based applications, backend servers, cloud services) and mHealth (mobile apps and mobile devices) resources such as Baobab Health Trust (http://baobabhealth.org/) and D-Tree International (https://www.d-tree.org/). While there is still a long way to go to make such technologies truly pervasive, it is clear from the initial findings that the use of eHealth has substantially improved data collection and overall operational activities within the Ministry of Health. Such progress will provide the necessary evidence to make a credible case to increase investment in medical technologies for this emerging healthcare system.

In summary, the experience gained by the students highlighted the utility of eHealth programmes in delivering healthcare services to remote parts of the country. Interchange of personnel between the Global North and South allows dissemination of resource-poor medical issues to a wider audience base in the medical field. We recommend that such initiatives are encouraged and believe that such initiatives are the basis of more concrete institutional collaborations between the Global North and the Global South.

## Conclusion

Innovation such as e-Health and low-cost biomarker development are key to improving life expectancy of people in remote areas of Malaŵi where there are no doctors and a paucity of other allied healthcare professionals. While Malaŵi remains a very poor nation with an ill-resourced healthcare system, pockets of North-South collaboration have led to an improvement in service delivery including the introduction of eHealth programmes which have been streamlining medical records and data collection for research programmes aimed at improving service delivery. The recent workshop in Cork brought together interested parties in improving Malaŵian healthcare systems including investment in postgraduate education. In order to consider further innovative eHealth projects, strategies and future directives for future funding were discussed, including potential grants from traditional grant-giving bodies, but also using corporate social responsibility directives of commercial companies active in Malaŵi from the manufacturing and banking sectors to develop a partnership with government to break the impasse currently seen.
